# Supplementary material for: Activation and evasion of type I interferon responses by SARS-CoV-2
Source: Nat Commun. 2020 Jul 30;11:3810. doi: 10.1038/s41467-020-17665-9 (PMC7392898; doi:10.1038/s41467-020-17665-9)
Supplement: Supplementary file 1 — Supplementary Information [file 41467_2020_17665_MOESM1_ESM.pdf]

SUPPLEMENTAL INFORMATION

Activation and Evasion of Type I Interferon Responses by SARS-CoV-2

Lei et al.

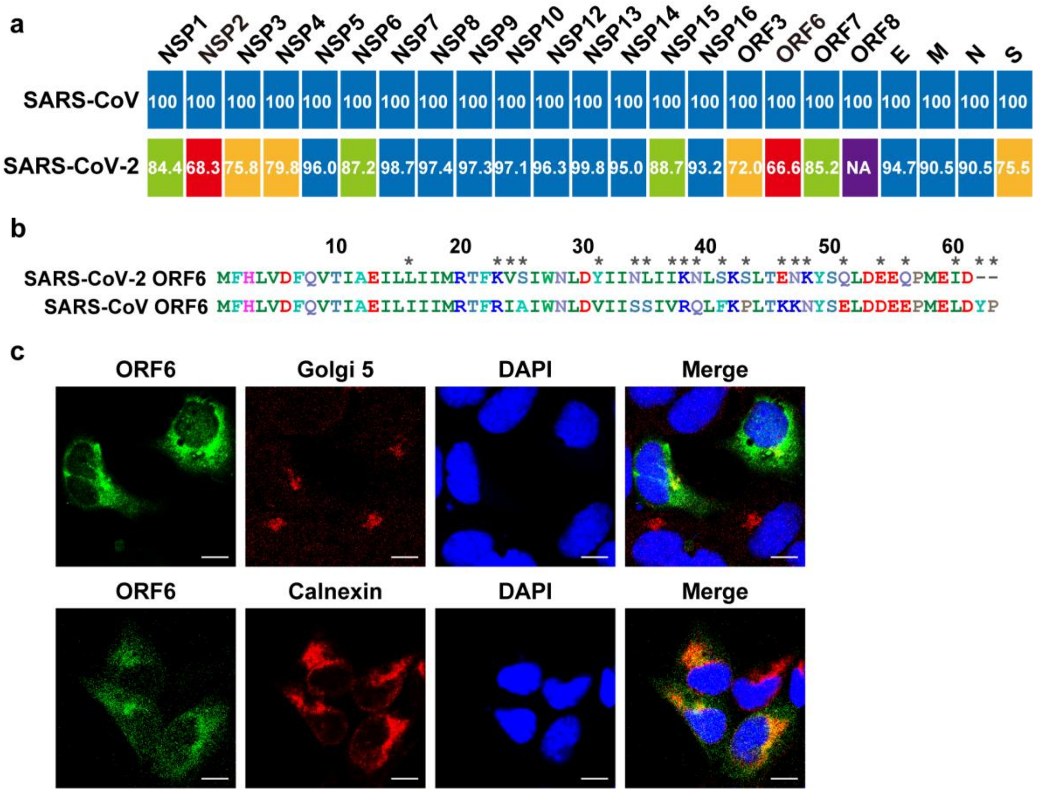

Supplementary Figure 1. Amino acid identity between SARS-CoV-2 and SARS-CoV proteins, and cellular distribution of ORF6. a Amino acid identity between proteins encoded by SARS-CoV and SARS-CoV-2. The identity value of all SARS-CoV proteins was set to 100. **b** Sequence alignment of SARS-CoV ORF6 and SARS-CoV-2 ORF6. **c** Cellular distribution of SARS-CoV ORF6. HEK293 cells were transfected with plasmids expressing Flag-tagged SARS-CoV-2 ORF6. After 24 h, cells were stained with anti-GolgiB1 (red), anti-calnexin (red) and anti-Flag (green) antibodies. Scale bar, 10 μm. All experiments were done at least twice, and one representative is shown.

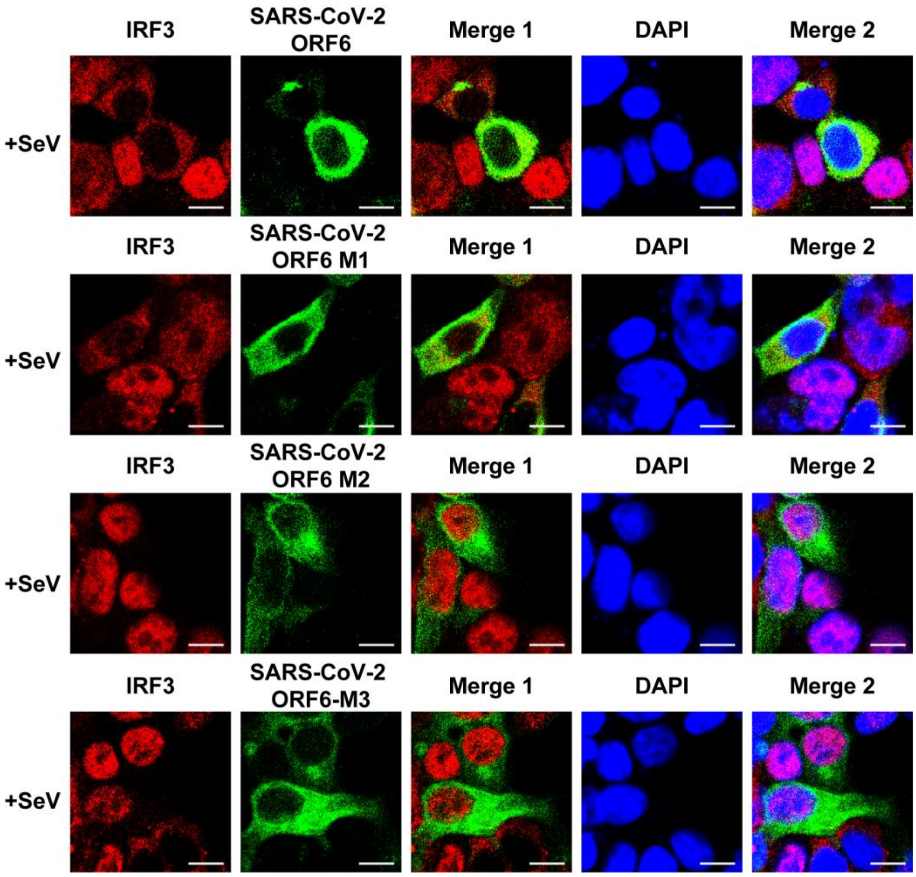

Supplementary Figure 2. ORF6 inhibits IRF3 nuclear translocation via its C-terminus. HEK293 cells were transfected with plasmids expressing Flag-tagged wildtype ORF6 or indicated ORF6 variants. At 24 h post-transfection, cells were infected with SeV for 4 h. Cells were then stained with anti-IRF3 (red), anti-Flag (green), and DAPI (blue). Merge 1 and Merge 2 indicate the merged red and green channels and the merged red, green, and blue channels, respectively. Scale bar, 10 μm. All experiments were done at least twice, and one representative is shown.

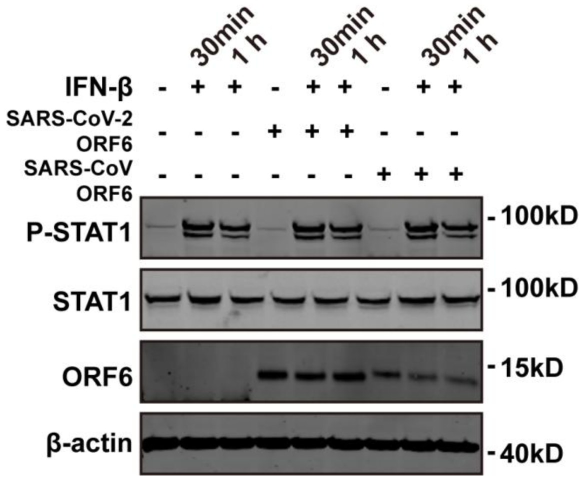

Supplementary Figure 3. ORF6 does not inhibit STAT1 phosphorylation in Vero cells. Vero cells were transfected a control plasmid, or with plasmids expressing SARS-CoV ORF6 or SARS-CoV-2 ORF6. At 24 h post-transfection, cells were treated with 1000 U/ml IFN-β for 30 min or 1 h. Phosphorylation of STAT1 was detected by Western blot using a phospho-STAT1-specific antibody. All experiments were done at least twice, and one representative is shown. Source data are provided as a Source Data file.

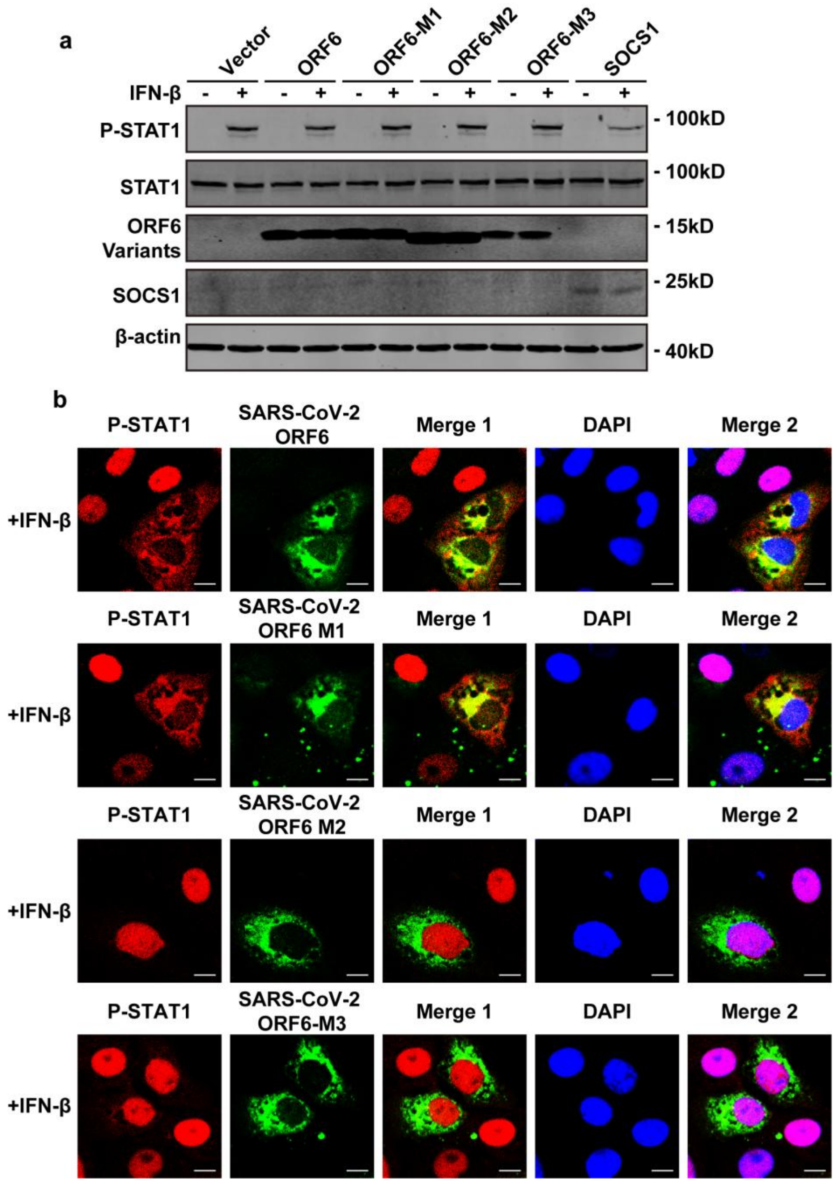

Supplementary Figure 4. The SARS-CoV-2 ORF6 or its variants does not inhibit STAT1 phosphorylation. a Effect of SARS-CoV-2 ORF6 and its variants on IFN-β-induced phosphorylation of STAT1. HEK293T cells were transfected with plasmids expressing wildtype ORF6 or indicated ORF6 variants, or with a plasmid expressing SOCS1. At 24 h post-transfection, cells were treated with 1000 U/ml IFN-β for 30 min. The phosphorylation of STAT1 was detected by Western blot. **b** Effect of SARS-CoV-2 ORF6 and its variants on nuclear translocation of phosphorylated STAT1. Vero cells were transfected with plasmids expressing Flag-tagged wildtype ORF6 or indicated ORF6 variants. At 24 h post-transfection, cells were treated with 1000 U/ml IFN-β for 30 min. Cells were then stained with anti-phospho-STAT1 (red), anti-Flag (green), and DAPI (blue). Merge 1 and Merge 2 indicate the merged red and green channels and the merged red, green, and blue channels, respectively. Scale bar, 10 μm. All experiments were done at least twice, and one representative is shown. Source data are provided as a Source Data file.

Supplementary Table 1 – Primers used in this study

Gene name	Primers	Reference
IFNβ	Forward primer<br>TAGCACTGGCTGGAATGAG<br>Reverse primer<br>GTTTCGGAGGTAACCTGTAAG	1
ISG56	Forward primer<br>TACAGCAACCATGAGTACAA<br>Reverse primer<br>TCAGGTGTTTCACATAGGC	1
ISG54	Forward primer<br>CTGCAACCATGAGTGAGAA<br>Reverse primer<br>CCTTTGAGGTGCTTTAGATAG	1
P gene of Sendai virus	Forward primer<br>CTCTGGGAGAACAAGCAAGC<br>Reverse primer<br>TCGCCCAGATCCTGAGATAC	2

1. Lei, X. et al. The 3C Protein of Enterovirus 71 Inhibits Retinoid Acid-Inducible Gene I-Mediated Interferon Regulatory Factor 3 Activation and Type I Interferon Responses. *J Virol* **84**, 8051–8061 (2010).
2. Liu, B. et al. The C-Terminal Tail of TRIM56 Dictates Antiviral Restriction of Influenza A and B Viruses by Impeding Viral RNA Synthesis. *J Virol* **90**, 4369–4382 (2016).
